# Polyorchidism during orchidopexy; A case report with review of literature

**DOI:** 10.1016/j.eucr.2021.101750

**Published:** 2021-06-11

**Authors:** Hiwote Girma Assefa, Assefa Mekonnen Sedeta, Hana Abebe Gebreselassie

**Affiliations:** St. Paul's Hospital Millennium Medical College, Department of Surgery, Addis Ababa, Ethiopia

**Keywords:** Polyorchidism, Triorchidism, Supernumerary testis, Orchidopexy

## Abstract

Polyorchidism is a rare congenital urologic abnormality. It is usually diagnosed incidentally by imaging or during surgery. We report a case of triorchidism, in a 2 years old boy which was discovered during elective orchidopexy. The testes was localized in the inguinal region and was of normal size. We opted for orchidopexy of this supernumeray testis due to its size and its ability to reach the scrotum. The preservation of the supernumerary testis has been a controversial issue. This case report offers brief discussion of polyorchidism with review of similar literature.

## Introduction

Polyorchidism is a rare congenial anomaly catheterized by the presence of more than two testes which can be either intra- or extra-scrotal in location. The most common variant of polyorchidism reported is triorchidism. To date, around 200 cases have been reported.

Although the exact etiology of polyorchidism is not known, the proposed mechanism is considered to be longitudinal or transverse division of the genital ridge possibly by development of peritoneal bands during early embryogenesis. The most commonly used classification system for polyorchidism is the Leung Classification which is based on the probable embryologic variation.

Majority of patients are asymptomatic and are incidentally diagnosed either via imaging or during surgical explorations for undescended testis, inguinal hernia, testicular torsion, hydrocele and malignancy. The management of polyorchidism is controversial but generally depends on multiple factors like the location of testis, the reproductive potential, size of the testicle and age.

We report a rare case of triorchidism in a two years old patient who was incidentally diagnosed during elective orchidopexy with review of similar cases in attempt to shade further light into the subject matter.

## Case summary

**Fig. 1:**
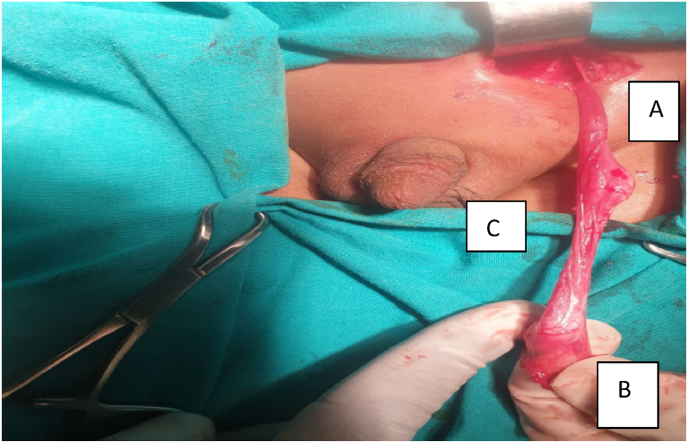
Left sided polyorchidism with common vas and separate epididymis, A cranial testis, B. Caudal testis C. Common vas.

A 2-year-old boy was diagnosed to have left undescended testis. On examination, right testis was palpable in the right hemi-scrotum but the left hemi-scrotum was empty with a palpable testis at the inguinal region. Since it was palpable, ultrasound was not done and patient was directly scheduled for orchidopexy. He was explored through left inguinal incision and the intra operative finding was, two non-atrophic testicles located in the inguinal region sharing the same vas and with two separate epididymis (Fig. 1). In this case, no biopsy was sent and both testicles were placed in the scrotum.

## Discussion

Polyorchidism is a rare congenital urogenital abnormality. It has been identified in variable age group ranging from the neonatal age to 8th decade of life but most patients are reported to be in their early adulthood.^1^

According to the Leung classification, there are 4 types of polyorchidism which are described as follows:

Type 1-Supernumerary testis that lacks an epididymis or vas and has got no attachment to the usual testis.

Type 2- the supernumerary testis draining into the epididymis of usual testis and sharing a common vas.

Type 3- the supernumerary testis has its own epididymis and both epididymis of ipsilateral testis drains into common vas.

Type 4- complete duplication of testis, epididymis and vas.

Type 2 is considered to be most common and together with type 3, they compromise 90% of polyorchidism.^2^ In our case, the patient had type 3 polyorchidism.

Most patients with polyorchidism are diagnosed incidentally on imaging or during surgical exploration. Polyorchidism is associated with undescended testicles in up to 40% of the cases.^3^ In our case, it was detected incidentally during exploration for undescended testis.

The most common type of polyorchidism is triorchidism and is mostly located on the left side (66%) which was also the case in our patient.^4^ As to the location of the supernumerary testis, the most common site is scrotal (66%), followed by inguinal (23%) and abdominal (9%).^2^ In our case, the supernumerary testis was located in the inguinal region.

The management of polyorchidism has been subjected to much debate. Due to current advances in imaging modalities, several authors recommend conservative management for patients with orthotopic polyorchidism while for patients with non-scrotal polyorchidism exploration is warranted. The other debatable issue is to either do orchidopexy or orchidectomy for the supernumerary testis. Those that are for orchidopexy, argue that preserving the supernumerary testis provides a better fertility potential and with the current advanced imaging modalities which are considered to be safe and highly sensitive for early detection of testicular malignancy, this mode of management is justified. The other authors claim that majority of the supernumerary testis have reduced or absent spermatogenesis and have increased risk of malignancy that warrants removal particularly in those which are atrophic.^5^

In addition, the incidence of testicular malignancy in polyorchidism in most reviews is said to be between 5.7 to 7%. This incidence rate is much higher than normal population (0.004%) and even higher than the incidence of cancer in undescended testis (0.045%). However, in all reported cancer cases, malignancy was found only in a non-scrotal (abdominal or inguinal) supernumerary testis.^5^

In our case, we have opted to preserve the non-atrophic testis which was placed in the scrotum. Moreover, we counseled the parents on the need for long-term surveillance.

## Conclusion

This case illustrates the unexpected presentation of polyorchidism during elective orchidopexy and the management controversies regarding the supernumerary testis. Hence, surgeons doing groin exploration should be aware of this condition.

## Declarations

Ethics approval and consent to participate:

Written informed consent was obtained from the parents for publication of this case series and accompanying images. A copy of the written consent is available for review by the Editor-in-Chief of this journal on request.

## Availability of data and materials

The datasets with more images and patient data are available from the corresponding author on reasonable request.

## Funding

This research did not receive any specific grant from funding agencies in the public, commercial, or not-for-profit sectors.

## Authors’ contributions

HGA and HAG were primarily involved in the management of the patient. AMS was also involved in the management of the patient and follow up. HAG wrote the case summary and the case report with review of the literature was written jointly by HAG and HGA. All authors have read and approved the final case report.

## Registration of research studies

Registry not required as this is not a first-in-man case report.

## Guarantor

Dr. Hiwote Girma Assefa.

Department of Surgery.

St. Paul's Hospital Millennium Medical Collage, Ethiopia.

## Provenance and peer review

Not commissioned, externally peer-reviewed.

## Declaration of competing interest

The authors declare that they have no competing interests.
